# A cellular system to study responses to a collision between the transcription complex and a protein‐bound nick in the DNA template

**DOI:** 10.1002/1873-3468.70053

**Published:** 2025-05-01

**Authors:** Petra Herring, Morten Roedgaard, Camilla Myrup Holst, Helene Christensen, Birgitta R. Knudsen, Lotte Bjergbaek, Anni Hangaard Andersen

**Affiliations:** ^1^ Department of Molecular Biology and Genetics Aarhus University Aarhus C Denmark; ^2^ Present address: Genau & More Aarhus N Denmark; ^3^ Present address: Fujifilm Diosynth Biotechnologies Hillerød Denmark

**Keywords:** camptothecin, collision, DNA topoisomerases, Flp recombinase, protein‐bound DNA nick, RNA polymerase II, ubiquitination

## Abstract

We present a transcription‐coupled Flp‐nick system enabling a stable protein‐bound nick mimicking a topoisomerase I–DNA cleavage complex. The nick is introduced at a single site within a controllable *LacZ* gene inserted into the *Saccharomyces cerevisiae* genome. This system allows unique single‐site studies of a frequently occurring damage within a transcription unit *in vivo*. As proof of principle, we demonstrate RNA polymerase II accumulation at the damage site when MG132 inhibits the proteasome. Similarly, accumulation occurs when polymerase ubiquitination is abolished by deletion of the ubiquitinase *ELC1* gene. This indicates that a topoisomerase I–DNA mimicking cleavage complex *per se* induces RNA polymerase II ubiquitination and degradation. These findings advance understanding of cellular responses to topoisomerase I‐targeting drugs used in cancer chemotherapy.

## Graphical abstract

We present the cellular transcription‐coupled Flp‐nick system allowing the introduction of a Top1‐mimicking cleavage complex (Flpcc) at a Flp recognition target site within a controllable *LacZ* gene. *LacZ* transcription leads to the collision of RNA polymerase II (RNAPII) with Flpcc, and this causes RNAPII stalling, ubiquitination, and degradation. This system enables detailed studies of RNAPII destiny after collision and repair processes, with or without transcription.

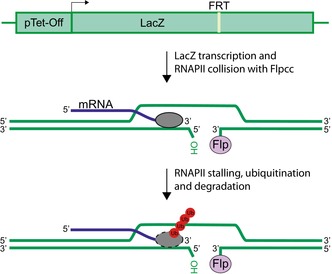

## Abbreviations

**ARS**, Autonomously replicating sequence

**ChIP**, Chromatin immunoprecipitation

**Chr**, Chromosome

**FACS**, Fluorescence‐activated cell sorting

**Flp**, Flippase

**Flpcc**, Flp‐DNA cleavage complex

**FRT**, Flp recognition target

**GAPDH**, Glyceraldehyde 3‐phosphate dehydrogenase

**HA**, Human influenza hemagglutinin

**MATa**, Maiting type a

**PCR**, Polymerase chain reaction

**RNAPII**, RNA polymerase II

**RT‐qPCR**, Real‐time quantitative PCR

**SDS**, Sodium dodecyl sulfate

**TC Flp‐nick**, Transcription‐coupled Flp‐nick

**Top1**, Topoisomerase I

**Top1cc**, Topoisomerase I‐DNA cleavage complex

**UV**, Ultraviolet light

**WB**, Western blot

**YP**, Yeast peptone

**YPD**, Yeast extract peptone dextrose

The ability of cells to transcribe their genomes is a prerequisite for the survival of all organisms. Even small temporal perturbations in gene expression can have dramatic consequences for the cell. Different types of DNA damages are known to cause RNA polymerase II (RNAPII) stalling [1]. Stalling is toxic to the cells as it inhibits further transcription of the gene holding the damage. Furthermore, the damage, if unrepaired, can block subsequent replication cycles with a huge impact on genomic stability [2, 3]. The existence of efficient pathways that ensure repair of damages from active genes as well as pathways that take care of the stalled RNAPII is therefore crucial. Most *in vivo* studies investigating how cells respond to DNA damage within active genes have been conducted using radiation and chemical agents to induce damage [4, 5, 6, 7]. The damage generated in this way is introduced genome‐wide and is often associated with the production of secondary damage, which complicates an unraveling of the cellular responses to the primary damage.

Here we describe the transcription‐coupled Flp‐nick (TC Flp‐nick) system, where an irreversible protein‐bound nick is created at a unique site within a controllable transcription unit in the *Saccharomyces cerevisiae* genome. The system, which is based on our earlier presented Flp‐nick system [8], allows studies of the events occurring when RNAPII encounters a protein‐bound nick. It takes advantage of the Flp recombinase, which catalyzes a single‐stranded cleavage‐religation reaction. The cleavage reaction is accomplished by a nucleophilic attack from an active site tyrosine on the phosphodiester backbone of the DNA [9]. In this process, a single‐stranded break is formed, which has a free 5′‐hydroxyl end and Flp covalently attached to the 3′‐end. The TC Flp‐nick system utilizes a modified Flp recombinase, FlpH305L, which is able to perform the cleavage reaction, but does neither accomplish the subsequent strand exchange nor the religation reaction [10]. The enzyme therefore creates long‐lived Flp‐DNA cleavage complexes (Flpccs). In terms of the creation of a covalent 3′‐phosphotyrosyl cleavage intermediate, Flp‐mediated cleavage resembles cleavage performed by DNA topoisomerase I (Top1). Top1 is a ubiquitous enzyme, which relieves topological stress in the DNA arising during transcription and replication [11]. The enzyme changes DNA topology by breaking and religating one strand of the DNA, during which a transient Top1‐DNA cleavage complex (Top1cc) is formed [12]. Top1ccs have attracted much attention, since treatment of cells with camptothecin analogs transforms these into long‐lived complexes, which eventually kill the cell [13]. Camptothecin analogs are therefore used as chemotherapeutic agents [14, 15]. Flpccs mimic the long‐lived camptothecin‐stabilized Top1ccs. In contrast to cleavage by Top1, Flp‐mediated cleavage is site‐specific, that is, the enzyme only cleaves DNA at the Flp recognition target sequence (FRT) [9]. The exact number and positions of Flpccs can therefore be controlled by inserting the target sequence into the genome at selected positions. Furthermore, the timing of Flpcc formation can be controlled by integrating FlpH305L under the control of an inducible promoter. Much can therefore be learned about how the cell deals with Top1ccs from studies of Flpcc [8, 16, 17].

In the earlier published Flp‐nick system, the FRT site was inserted upstream to the early firing origin *ARS607* in a nontranscribed region. Upon formation of Flpcc at the FRT site, cells were released into the S phase to allow studies of the cellular responses to a collision between the DNA replication complex and the Flpcc [8].

In the TC Flp‐nick system, a single FRT site has been integrated inside the open reading frame of a LacZ gene placed under the control of the Tet‐Off promoter in the *S. cerevisiae* genome. FlpH305L is expressed under the control of the *GAL10* promoter. Thus, induction of FlpH305L by galactose results in a single protein‐bound nick inside the LacZ transcription unit. Since the exact location and timing of the protein‐bound nick are known, a wide range of biochemical assays can be utilized to study the cellular responses to the collision between the transcription complex and the protein‐bound nick. As a Proof of Principle, we here demonstrate how advantage can be taken of the TC Flp‐nick system to investigate the destiny of RNAPII upon collision with the damage.

## Materials and methods

### Plasmids, yeast strains, growth conditions, and FACS analysis

All *S. cerevisiae* strains used are derivatives of W303a, and have, besides the indicated mutations, the following genotype: MATa *ade2*‐1 *trp1*‐1 *his3*‐11 *his3*‐15 *ura3*‐1 *leu2*‐3 *leu2*‐112 *canI*‐100 Cir0. An overview of the yeast strains used in this study is shown in Table 1. The TC Flp‐nick strain (Ay‐366) was constructed by linearizing the plasmid AC403 with EcoRV and transforming it into LBy‐448. AC403 contains the LacZ gene under the control of the Tet‐Off promoter with an FRT site inserted in the unique DraIII site within the LacZ open reading frame. Furthermore, the plasmid contains a sequence homologous to a genomic region located ∼3 kb upstream to ARS607 on chromosome VI in the *S. cerevisiae* genome. This sequence holds the unique EcoRV site used for linearization. The plasmid contains the KanMX selection marker. Gene disruptions were made using pFa6a PCR‐based cassettes and subsequently verified by PCR, sequencing, and phenotypic analyses. Other derivatives were made by standard techniques of mating and tetrad dissection. For most experiments, yeast strains were grown at 30 °C in YP medium with 2% raffinose. In experiments, where MG132 was used, cells were grown in synthetic media containing SDS and with proline as the sole nitrogen source to enhance the uptake of MG132 [18]. Galactose was added to a final concentration of 3% for the induction of FlpH305L. The cells were arrested in G1 of the cell cycle with 4 μg·mL^−1^ α‐factor (Lipal Biochem, Zürich, Switzerland), and arrest was verified by FACS analysis. For this, 0.25 × 10^7^ cells were fixed overnight in 70% ethanol at 4 °C. After centrifugation, 200 μg·mL^−1^ RNase A (Sigma, MA, USA) in 50 mm Tris‐HCl at pH 7.4 was added for 2 h at 37 °C. Cells were spun and resuspended in 10 μg·mL^−1^ propidium iodide (Sigma) in 50 mm Na‐citrate (pH 7.0) and incubated overnight in the dark at 4 °C. Samples were analyzed using a FACSCalibur (Becton Dickinson, Franklin Lakes, NJ, USA) or a CytoFlex (Beckman Coulter Life Sciences, CA, USA).

**Table 1:** Yeast strains used in this study.

Strain	Name	Genotype	Assays	Source
LBy‐448	Flp3xHA	*LEU2‐GAL10‐FlpH305L‐3xHA‐HIS3*	Background strain to create the TC Flp3xHA‐nick system	[8]
Ay‐366	TC Flp3xHA‐nick	*LEU2‐GAL10‐FlpH305L‐3xHA‐HIS3*, *KanMX‐Tetoff‐LacZ::FRT*	Used in Fig. 2B–D,F and Figs S1, S2A	This study
Ay‐430	TC Flp‐nick	*LEU2‐GAL10‐FlpH305L*, *KanMX‐Tetoff‐LacZ::FRT*	Used in Figs 2E, 3A–D and Figs S1, S2B	This study
Ay‐451	TC Flp‐nick *elc1Δ*	*LEU2‐GAL10‐FlpH305L*, *KanMX‐Tetoff‐LacZ::FRT*, *elc1::TRP1*	Used in Fig. 3D and Fig. S2B	This study
Ay‐464	TC Flp	*LEU2‐GAL10‐FlpH305L*, *KanMX‐Tetoff‐LacZ*	Used in Figs 2E, 3A–C and Fig. S2B	This study

### RT‐qPCR

For analysis of transcription levels, cells were grown as described above, and samples of 2 × 10^7^ cells were collected at the indicated time points and stored at −80 °C until use. RNA was purified with RNeasy (Invitrogen, Carlsbad, CA, USA), and cDNA was made by QuantiTect Reverse Transcription Kit (Qiagen, Germany). Real‐time PCR was performed with HOT FIREPol EvaGreen qPCR Mix Plus (ROX) qPCR kit (Solis Biodyne, Tartu, Estonia) and used to quantify mRNA levels, using a Stratagene MX3000 (Agilent, Santa Clara, CA, USA). Fold change was calculated using the 2^−ΔΔCt^‐method with actin or the mean of actin and GAPDH as reference as indicated in the Figure legends. The forward and reverse primers used for RT‐qPCR of Flp, LacZ, GAPDH, and Actin are TGGGAAATTGGAGCGATAAG and CTGCCACTCCTCAATTGGAT, CGAATACCTGTTC CGTCA and ACTGTTTACCTTGTGGAGC, CACCAACTGTTTGGCTCCAT and TAGCAGCA CCGGTAGAGGAT, and GCCTTCTACGTTTCCATCCA and GGCCAAATCGATTCTCAAAA, respectively.

### ChIP and western blotting

ChIP was performed using either monoclonal antibodies against HA (Santa Cruz, CA, USA) to precipitate HA‐tagged FlpH305L (FlpH305L3xHA) or against the RPB1 subunit of RNAPII (ab5408 available from Abcam, Cambridge, UK). ChIP was performed on 2.5 × 10^8^ cells as previously described [8]. As the Flp recombinase forms long‐lived covalently linked protein‐DNA complexes upon cleavage, the formaldehyde cross‐linking step was omitted in ChIP experiments of FlpH305L3xHA [8]. Furthermore, for ChIP of RNAPII, an extra washing step was used as previously described [19]. The forward and reverse primers used for ChIP of RNAPII at the 3′‐end of LacZ (P1), of RNAPII and FlpH305L at the FRT site in LacZ (P2), of RNAPII in the middle of the coding region in *PHO87* and *PSE1*, or of FlpH305L at the telomere on the right arm of chromosome VI are CGAATACCTGTTCCGTCA and ACTGTTTACCTTGTGGAGC, TGATTGAAGCAGAAGCC TG and TTAACGCCTCGAATCAGC, AGGGTACTAAAAGACGATGA and TTATATTG CGACAAGGCTTC, CAACCGAATGGAGAGAAAGA and GATGAGGATCGTTGATGAGG, and CCATAATGCCTCCTATATTTAGCCTTT and TCCGAACGCTATTCCAGAAAGT, respectively.

Immunoprecipitated DNA was quantified by real‐time PCR using HOT FIREPol EvaGreen qPCR Mix Plus (ROX) qPCR kit (Solis Biodyne, Tartu, Estonia) on a Stratagene MX3000 (Agilent). All samples were measured in duplicates or triplicates to minimize technical variation.

Western blotting of FlpH305L3xHA was performed with standard procedures using monoclonal antibodies targeting the HA‐tag (Santa Cruz, CA, USA).

### Southern blotting

Cells were grown as described above, and samples of 1.5 × 10^7^ cells were collected and stored at −80 °C until use. Genomic DNA was isolated using Genomic tip 20/G (Qiagen, Germany). DNA was digested with SacI, and samples were analyzed on denaturing alkaline gels, followed by transfer to a membrane and hybridization to a radioactive labeled probe. A marker containing DNA of 1400 and 2200 bp was included in the gel to verify sizes of detected DNA fragments.

## Results

### A cellular system to study the responses to a collision between the transcription complex and a protein‐bound DNA nick

In order to create an *in vivo* system allowing us to study the consequences of a collision between the transcription complex and a protein‐bound DNA nick in *S. cerevisiae*, we constructed the TC Flp‐nick system. The system is a modified version of the Flp‐nick system, which we published previously [8]. Both systems take advantage of the step‐arrest mutant of the Flp recombinase (FlpH305L). The mutant enzyme specifically recognizes and cleaves the FRT sequence forming an irreversible Flpcc at this site mimicking Top1cc [9].

**Fig. 1:**
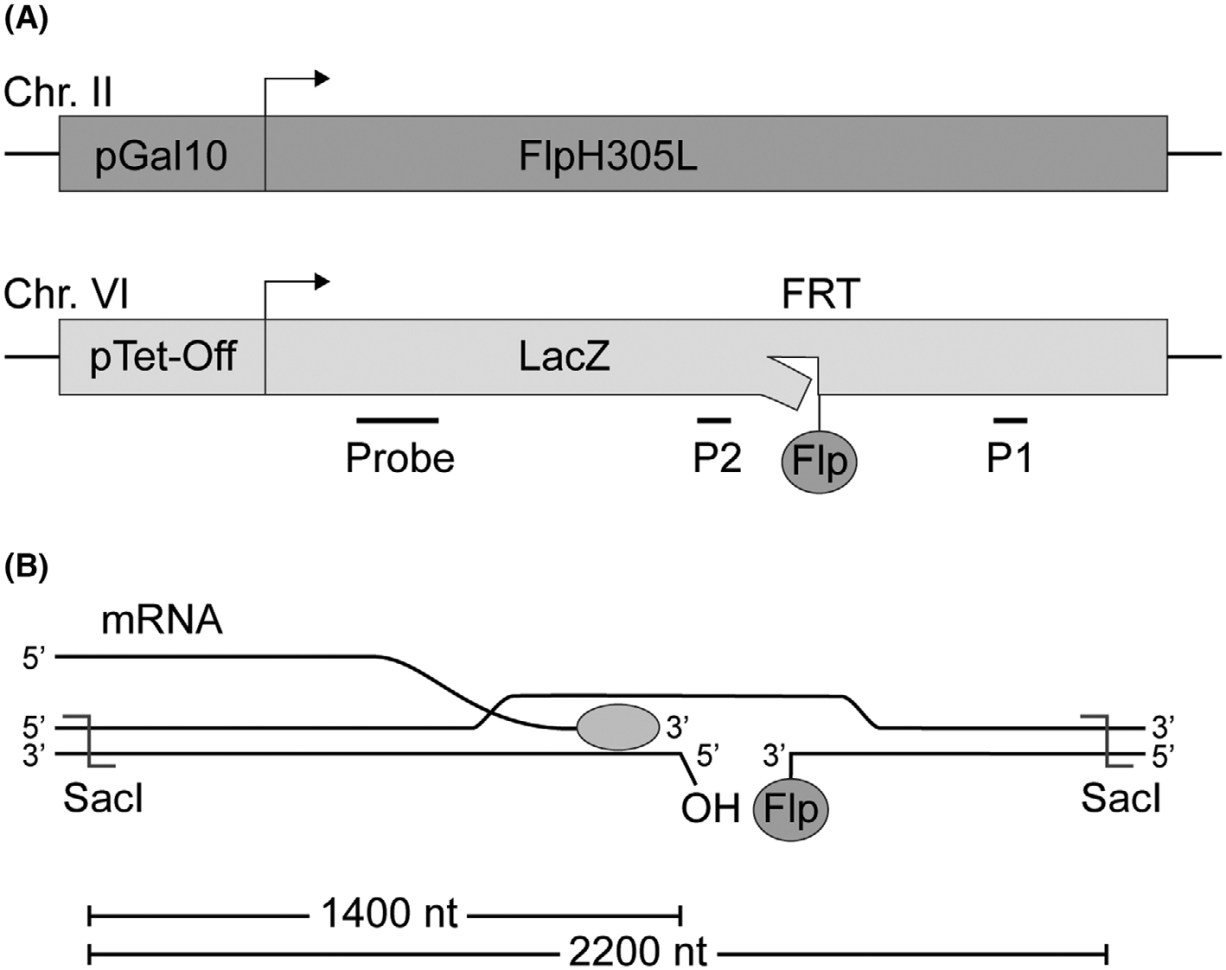
Schematic illustration of the TC Flp‐nick system and the collision of the transcription complex with Flpcc. (A) Schematic overview of the TC Flp‐nick system. Upon galactose treatment, TC Flp‐nick cells express FlpH305L which binds to the FRT site, here shown by cleavage on the template strand in the inserted LacZ gene. LacZ is transcribed in the absence of doxycycline and repressed in the presence of doxycycline. Positions of probes used in Southern blots and primer sets used in ChIP (P1 and P2) are indicated. Chr., chromosome. (B) Illustration of collision between RNAPII and Flpcc. The SacI cleavage sites as well as sizes of products obtained upon Flp‐mediated cleavage are indicated. The transcription complex is indicated by a gray ellipse.

To construct the TC Flp‐nick system, we used a yeast strain having the sequence encoding FlpH305L expressed from the inducible *GAL10* promoter inserted on chromosome II (Fig. 1A). The FRT sequence was inserted in the open reading frame of the LacZ gene in such a way that a major Flp‐mediated cleavage site is found on the template strand and a minor site is found on the nontemplate strand [8]. The modified gene under control of a Tet‐Off promoter was next integrated on chromosome VI with the FRT sequence located ∼3 kb upstream to ARS607 (Fig. 1A). Thus, in the presence of galactose, FlpH305L is expected to cleave at the FRT site in the LacZ gene, forming an irreversible Flpcc with Flp covalently linked to the 3′ end of the cleaved DNA strand. In the absence of doxycycline, the Tet‐Off promoter is active, and in cells having a Flpcc on the template strand, the RNAPII will encounter the protein‐bound nick from the protein‐free side (Fig. 1B).

The TC Flp‐nick system was created either with or without a 3xHA‐tag on FlpH305L. The strain holding the tagged version (TC Flp3xHA‐nick) was generated to allow FlpH305L ChIP‐ and WB analyses. As tagging reduces Flp activity/stability (Fig. S1), experiments were conducted with the strain without an HA‐tag (TC Flp‐nick) unless otherwise indicated (Table 1).

### Verification of the TC Flp‐nick system

**Fig. 2:**
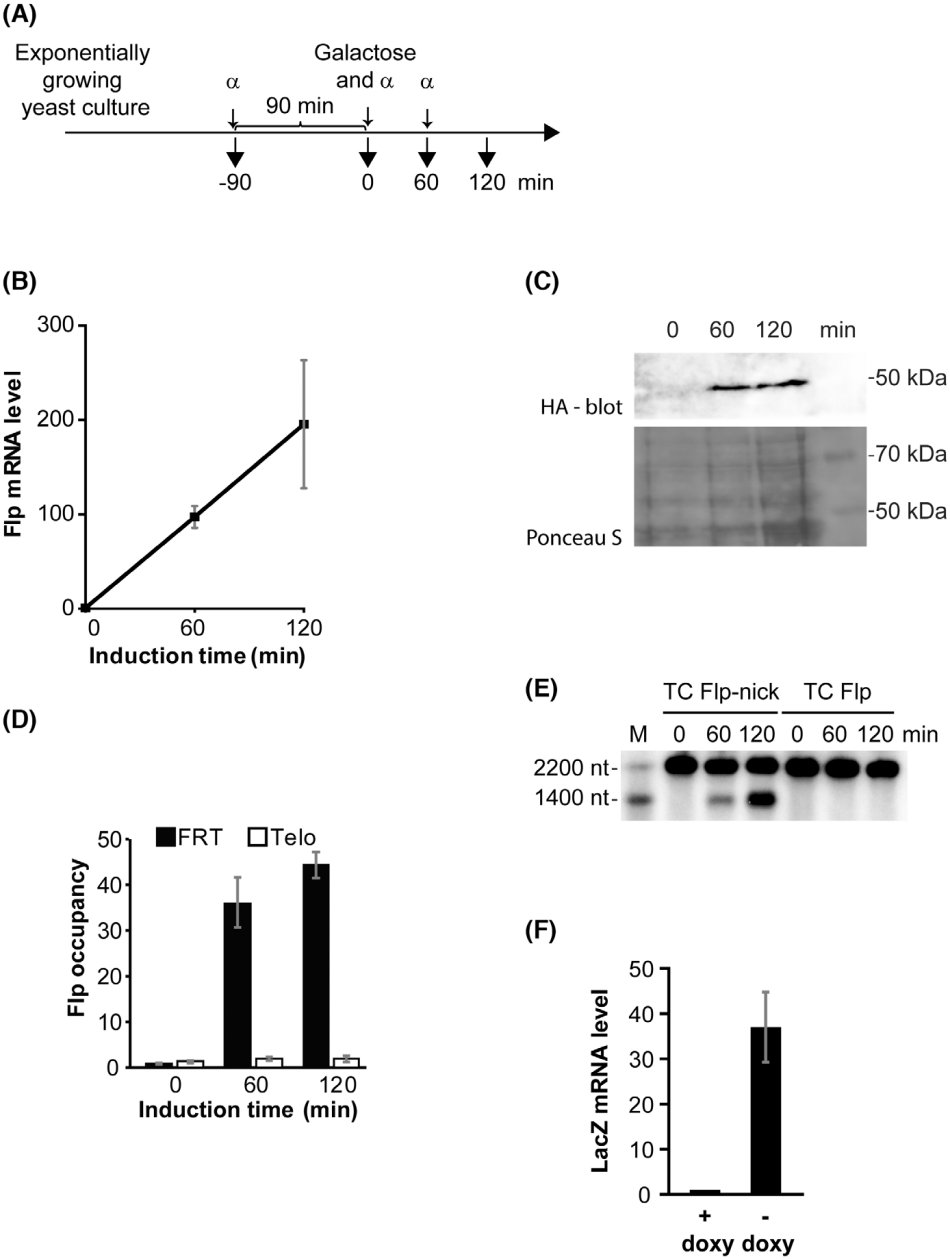
Verification of the TC Flp‐nick system. (A) Experimental setup. Yeast cells were grown to the exponential phase in raffinose media in the absence of doxycycline (unless otherwise stated) and arrested in G1 by α‐factor. Galactose was added to induce FlpH305L, and samples were collected at the indicated time points. α, α‐factor. The TC Flp3xHA‐nick strain was used except in (E), where TC Flp‐nick and TC Flp were used. (B) FlpH305L mRNA levels 0, 60, and 120 min after addition of galactose. qPCR was performed on total RNA purified from individual samples. Data were normalized to mean values of GAPDH and Actin as described in the ‘Materials and Methods’ section. Error bars, SD (*n* = 3). (C) Western blot to detect FlpH305L protein levels 0, 60, and 120 min after addition of galactose. Flp protein was visualized by antibodies targeting the HA‐tag on FlpH305L (*Upper Panel*). A Ponceau stain of the membrane is shown as a loading control (*Lower panel*). (D) ChIP analysis to measure FlpH305L occupancy at the FRT site. Samples were subjected to ChIP using antibodies against the HA‐tag on FlpH305L and primer set P2. A primer set recognizing the right arm telomere of chromosome VI was used as a control. The formaldehyde cross‐linking step was omitted due to covalent binding of Flp to DNA after cleavage. FlpH305L enrichment was calculated as immunoprecipitated material relative to beads alone. Error bars, SD (*n* = 3). (E) Southern blot to detect FlpH305L‐mediated cleavage at the FRT site. Cells with (TC Flp‐nick) or without (TC Flp) an FRT site inside LacZ were treated as outlined in (A). Genomic DNA was isolated from samples, digested with SacI, and exposed to alkaline gel electrophoresis and Southern blotting to visualize single‐stranded cleavage. M, DNA marker showing positions of the 1400 nt and 2200 nt fragments; nt, nucleotides. (F) LacZ mRNA levels in cells grown in the absence and presence of 1 mm doxycycline. The cells were grown overnight with or without 1 mm doxycycline and treated with α‐factor as indicated in (A). After 90 min in α‐factor, RNA was purified from the cells and subjected to qPCR using LacZ‐specific primers. Data were normalized to Actin. Error bars, SD (*n* = 3).

The TC Flp‐nick system was verified using the experimental setup demonstrated in Fig. 2A. Samples were withdrawn from an exponentially growing yeast culture at different time points after induction of FlpH305L by galactose. Doxycycline was avoided unless LacZ transcription should be turned off. To avoid complications from the collision of the DNA replication apparatus with the protein‐bound nick, studies were performed with yeast cells maintained in the G1 phase of the cell cycle. α‐factor was therefore added to exponentially growing yeast cells before induction of FlpH305L and at regular intervals after. FACS analyses were performed to verify G1 arrest (Fig. S2A).

To examine the level of FlpH305L expression, RNA was extracted from samples and analyzed by RT‐qPCR. An ~200‐fold increase in FlpH305L mRNA levels was revealed 2 h after the addition of galactose (Fig. 2B). Western blotting with antibodies against an HA‐tag fused to the C‐terminal end of FlpH305L revealed a corresponding increase in FlpH305L protein level upon galactose induction (Fig. 2C).

To investigate the binding of FlpH305L to the FRT site in the LacZ gene, chromatin immunoprecipitation (ChIP) analysis was performed with antibodies against the HA‐tag on Flp. The addition of galactose to the growth media caused an ~45‐fold enrichment of FlpH305L at the FRT site after 2 h, while no enrichment was observed at a control telomeric region (Fig. 2D). Thus, FlpH305L forms a stable complex at the FRT site.

As covalent linkage of the Flp enzyme is preceded by DNA cleavage, the ChIP experiment strongly suggested that Flp‐mediated DNA nicking had taken place at the FRT site. To confirm this, DNA isolated from samples collected after the addition of galactose was analyzed by alkaline gel electrophoresis after digestion with SacI. The restriction enzyme generates a 2200 bp DNA fragment holding the FRT site (Fig. 1B), which will give rise to cleavage products of 1400 and 800 nucleotides upon Flp‐mediated cleavage, where only the 1400 nucleotide product will be visible with the used probe. The cleavage product was observed in samples taken after galactose treatment, but not in samples taken before induction of the Flp enzyme (Fig. 2E). Furthermore, the cleavage product was absent in samples taken from yeast cells having the TC Flp‐nick system except that no FRT site was present in the LacZ gene (termed TC Flp). Taken together, FlpH305L specifically recognizes the inserted FRT site and generates a protein‐bound nick upon DNA cleavage.

Finally, RT‐qPCR was used to measure the levels of LacZ mRNA in cells grown in the presence and absence of doxycycline to measure the strength of the Tet‐Off promoter in the TC Flp‐nick system. A nearly 40‐fold reduction in LacZ mRNA levels was observed upon treatment with doxycycline (Fig. 2F), demonstrating that LacZ transcription is controllable in yeast cells harboring the TC Flp‐nick system.

Based on the presented verifications, the TC Flp‐nick system is applicable for studies of the consequences of a collision between the transcription complex and a protein‐bound nick.

### Collision between RNAPII and an induced Top1‐DNA mimicking cleavage complex results in ubiquitination and degradation of the polymerase

**Fig. 3:**
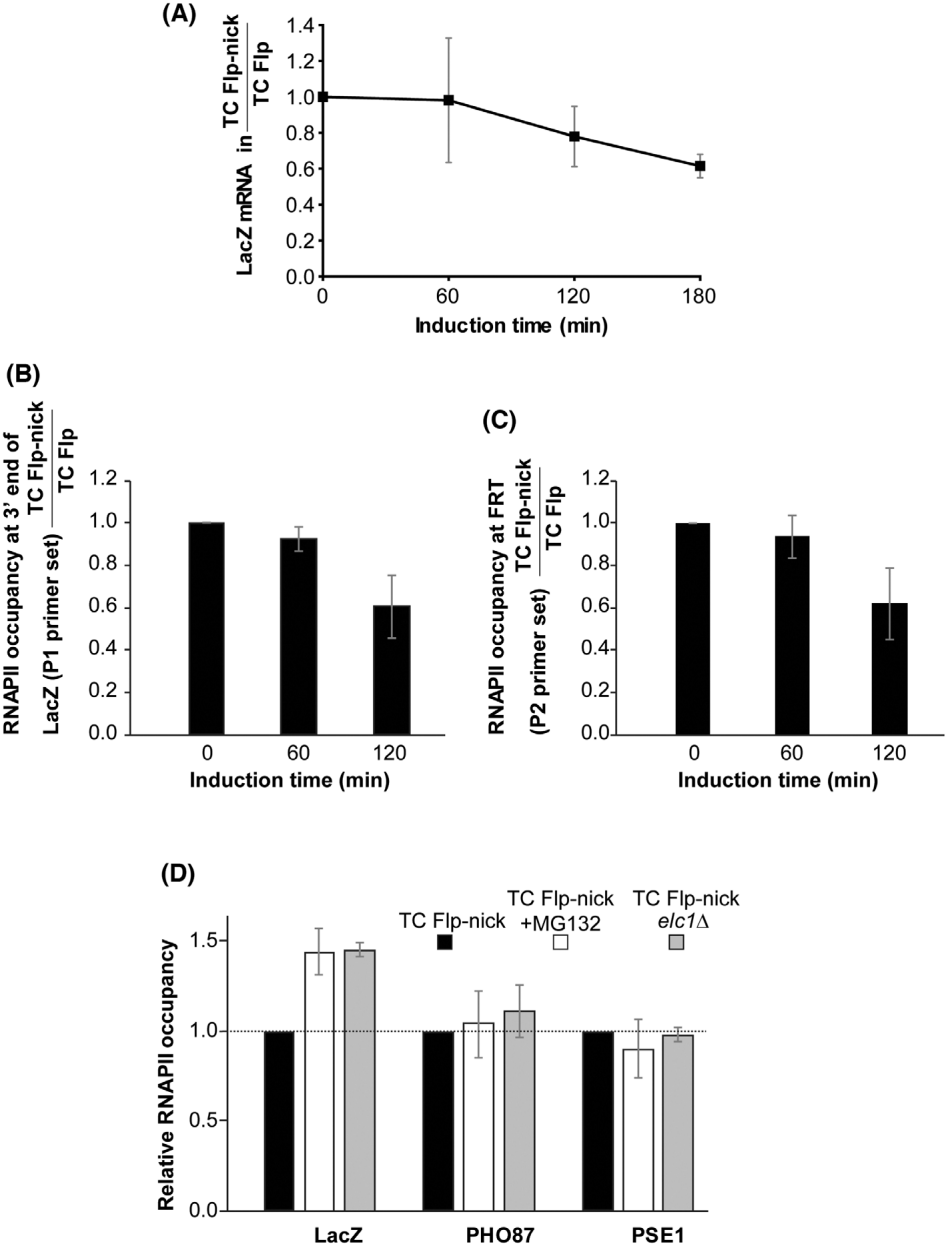
Effects of RNAPII collision with Flpcc (A) Influence of Flpcc on LacZ mRNA levels. TC Flp‐nick and TC Flp cells were treated as outlined in Fig. 2A except that α‐factor also was added 120 min after galactose induction to keep cells in G1. Total RNA was purified at the indicated time points and LacZ mRNA levels determined by qPCR using LacZ‐specific primers. Data were normalized to Actin. The level obtained in TC Flp‐nick cells was divided by the level obtained in TC Flp cells at the indicated time points, and the ratio at time point 0 was set to 1. Error bars, SD (*n* = 3). (B) ChIP analysis to measure the influence of Flpcc on RNAPII occupancy in the 3′ end of LacZ. TC Flp‐nick and TC Flp cells were treated as outlined in Fig. 2A, and ChIP was performed on formaldehyde cross‐linked samples using antibodies against RNAPII and primer set P1. RNAPII occupancy was calculated as described in the ‘Materials and Methods’ section. The occupancy in the TC Flp‐nick strain was divided by the occupancy in the TC Flp strain, and the ratio at time point 0 was set to 1. Error bars, SD (*n* = 3). (C) ChIP analysis to measure the influence of Flpcc on RNAPII occupancy at FRT. The experiment was performed as in (B) except that ChIP was performed with primer set P2. Error bars, SD (*n* = 3). (D) ChIP analysis to measure RNAPII occupancy at FRT in TC Flp‐nick cells treated with or without MG132 and in TC Flp‐nick *elc1∆* cells. Cells were treated as outlined in Fig. 2A except that cells with or without MG132 were grown in synthetic media containing SDS and proline as the sole nitrogen source. Samples were collected before and 120 min after addition of galactose, and ChIP was performed after formaldehyde cross‐linking using antibodies against RNAPII. RNAPII occupancy was measured in LacZ using primer set P2 and in two control genes, *PHO87* and *PSE1*. The relative RNAPII occupancy was calculated as the difference in RNAPII occupancy before and after galactose treatment normalized to this difference in the untreated wild‐type TC Flp‐nick strain (set to 1). Error bars, SD (*n* = 3).

To demonstrate the utility of the TC Flp‐nick system, we have studied the consequences of a collision between RNAPII and the induced Flpcc damage on transcription and for the RNAPII enzyme *per se*. For this purpose, we first used RT‐qPCR to compare LacZ mRNA levels obtained in cells with (TC Flp‐nick) or without (TC Flp) an FRT site in the LacZ gene after induction of FlpH305L by galactose treatment. The experimental setup was as illustrated in Fig. 2A. G1 arrest for the used strains was verified by FACS analyses (Fig. S2B). Only cells having a Flpcc on the template strand were expected to be affected in LacZ transcription. The presence of FRT resulted in a decrease in LacZ mRNA levels. The decrease became more pronounced with increasing FlpH305L expression (Fig. 3A), resulting in an ∼40% reduction 3 h after the addition of galactose. Thus, the formation of the protein‐bound nick interferes with mRNA formation.

To further investigate this, we used ChIP with P1 primers (Fig. 1A) to measure RNAPII density at the 3′‐end of LacZ in the two strains during FlpH305L induction. The ChIP assay revealed a decrease of approximately 40% in RNAPII density at the 3′‐end in the TC Flp‐nick strain relative to the TC Flp strain after 2 h in galactose (Fig. 3B). In correlation with the effect of the protein‐bound nick on LacZ mRNA production, the result suggests that a protein‐bound nick inside the open reading frame of a gene interferes with the progression of RNAPII as has been observed earlier for Top1cc introduced on the template strand in an *in vitro* transcription system [20]. The data strongly suggest that Flpcc affects RNAPII and transcription in the same way as Top1cc.

Studies of the collision between RNAPII and different barriers in the DNA have demonstrated backtracking or ubiquitination and proteasomal degradation of RNAPII after stalling [19, 21, 22, 23]. Furthermore, RNAPII stalling and proteasomal degradation have been observed in mammalian cell lines treated with camptothecin, which increases the amount of Top1ccs in the cell [24, 25, 26]. However, in studies using camptothecin, it is not possible to determine if stalling and proteasomal degradation take place due to a collision of RNAPII with Top1ccs or with DNA supercoils generated under these conditions. The level of Top1 and thus the level of supercoiling is expected to be unaffected in yeast strains harboring the TC Flp‐nick system. The system is therefore ideal for a study of the effect on RNAPII of a direct collision with a Top1‐DNA mimicking cleavage complex. If RNAPII backtracks/stalls without being degraded after encountering Flpcc, an increase in RNAPII occupancy was expected at this position in the LacZ gene. In contrast, a reduction in RNAPII occupancy was expected if RNAPII dissociates or is degraded. To discriminate between these two possibilities, we applied ChIP analysis to measure the RNAPII occupancy immediately upstream to FRT in the TC Flp‐nick and TC Flp strains using the P2 primers (Fig. 1A). The ChIP assay revealed a reduction in RNAPII occupancy at the FRT site in LacZ (Fig. 3C), indicating that RNAPII is degraded or dissociates from the DNA after it has encountered the protein‐bound nick.

To examine whether the reduction in RNAPII occupancy was due to proteasome‐mediated degradation, we first measured the change in RNAPII occupancy before and 120 min after galactose addition in TC Flp‐nick cells treated with or without the proteasome inhibitor MG132. Addition of MG132 caused a significant increase in RNAPII occupancy at the FRT site in the LacZ gene (Fig. 3D). In contrast, no increase in RNAPII occupancy was observed, when two control genes, *PHO87* and *PSE1*, of approximately the same length as LacZ were investigated in the same cells. This indicates that stalling of RNAPII at the protein‐bound nick provokes proteasomal degradation of the polymerase. Since proteasomal degradation of a protein in general is preceded by ubiquitination of the protein of interest [27], we next measured the change in RNAPII occupancy upon galactose induction in a wt TC Flp‐nick strain relative to a TC Flp‐nick strain lacking the ubiquitinase Elc1. Elc1 has earlier been demonstrated to catalyze RNAPII ubiquitination [28]. Similar to MG132, an increase in RNAPII occupancy was observed at the FRT site in TC Flp‐nick *elc1∆* cells, while no change was observed in the two control genes (Fig. 3D). Thus, Elc1 is important for RNAPII removal, strongly indicating that an encounter between RNAPII and Flpcc leads to ubiquitination of RNAPII before it is degraded by the proteasome.

Taken together, the obtained results demonstrate that the collision between RNAPII and a protein‐bound nick in the form of a Top1‐DNA mimicking cleavage complex causes an inhibition of RNAPII progression. The polymerase stalls and is subsequently removed via the ubiquitin‐proteasome degradation pathway. Furthermore, the results serve as Proof of Principle that the TC Flp‐nick system will be useful for future studies of the cellular consequences of a collision between the transcription complex and a protein‐bound nick.

## Discussion

Here we present the TC Flp‐nick system, which can be used to study the response mediated by the cell when the transcription machinery encounters a protein‐bound nick *in vivo*. The system has several advantages compared to studies using drugs or UV treatment, which induce global damage. Among the advantages are as follows: (1) the location of the damage is known; (2) only a single damage is generated; (3) the damage is inducible; and (4) the damage is generated within a controllable transcription unit. Thus, benefit can be taken of many biochemical assays, which are impossible or difficult to use, when damage is introduced globally. Furthermore, severe secondary effects, which influence the cellular responses, can be avoided when only one damage is present. In addition, the inducible nature of the damage makes it possible to investigate the impact of the damage in different phases of the cell cycle.

The TC Flp‐nick system allows at least two different kinds of studies. Due to the introduction of the damage in a transcription unit, the system permits an unraveling of the cellular pathways activated, when the transcription machinery encounters the damage, including pathways involved in modifications of RNAPII. Furthermore, as the damage mimics a drug‐stabilized Top1cc, the system can be used to identify repair pathways specifically associated with the repair of these complexes with and without transcription. In the present study, we have demonstrated the applicability of the TC Flp‐nick system to investigate what happens to RNAPII upon collision with a protein‐bound nick in yeast cells.

In the TC Flp‐nick system, FlpH305L‐mediated cleavage of the template strand at the FRT site in LacZ causes a collision between RNAPII and the protein‐bound nick, with RNAPII encountering the damage from the protein‐free 5′ OH end. Several scenarios can be envisioned when this happens. RNAPII may run off, it may backtrack to allow space for proper damage repair, it may stall to await repair of the damage, or it may be degraded to allow efficient repair [20, 25, 29]. These possibilities are not mutually exclusive. Stalling has been observed when RNAPII collides with Top1cc *in vitro* [20], and when it collides with other DNA damages [30, 31, 32, 33].

Several studies have demonstrated that RNAPII is degraded upon stalling. For example, RNAPII was shown to be ubiquitinated and degraded by the proteasome in cells with UV‐induced damage, where stalling occurred due to pyrimidine dimers [22, 31, 34, 35]. Degradation was also observed when RNAPII stalling was caused by head‐to‐head collision with another RNAPII [19], and in cells treated with camptothecin [21, 25]. In the latter case, degradation of RNAPII was suggested to take place due to Top1cc‐mediated stalling of the polymerase. However, as camptothecin also causes a depletion of the cellular Top1 level [20] leading to an accumulation of positive supercoils, which has been suggested to cause polymerase stalling [36], there are still some uncertainties as to what causes RNAPII stalling and degradation upon camptothecin treatment. In the present study, we have investigated how a single Flpcc affects transcription and the fate of RNAPII. We find that Flpcc inhibits transcription and blocks the progression of RNAPII. This is in line with the earlier observation that Top1cc introduced by camptothecin in an *in vitro* transcription system blocks RNAPII progression [20] and points to the similarity between Flpcc and Top1cc. Furthermore, RNAPII becomes degraded through the ubiquitin‐proteasome pathway when it encounters the damage. Since no change in superhelicity is expected with the TC Flp‐nick system, our studies favor the suggestion that camptothecin treatment leads to polymerase stalling and ubiquitination due to a collision of RNAPII with Top1ccs and illustrate the applicability of the TC Flp‐nick system to perform detailed studies of what happens when RNAPII encounters a protein‐bound nick. This will include studies of whether RNAPII backtracking occurs before degradation or if repair pathways involving RNAPII backtracking are the preferential choice if ubiquitination and proteasomal degradation is inhibited. Furthermore, the system may be helpful in unraveling the pathways used by the cell to differentiate between an RNA polymerase stalled due to DNA damage or paused during normal transcription [1]. Finally, as the system generates a single protein‐bound nick at a specific genomic position within a gene for which transcription is controllable, the system will allow comparison of pathways involved in the repair of the damage with and without transcription as well as studies of whether this type of damage causes checkpoint activation.

## Author contributions

PH and MR: design and conduction of experiments, manuscript writing. CMH and HC: conduction of experiments. BRK: resources, review, and editing. LB: conceptualization, design of experiments, resources, review, and editing. AHA: conceptualization, design of experiments, resources, and manuscript writing.

## Peer review

The peer review history for this article is available at https://www.webofscience.com/api/gateway/wos/peer‐review/10.1002/1873‐3468.70053.

## Supporting information


**Fig. S1.** The 3xHA tag reduces the cleavage activity/stability of FlpH305L.

**Fig. S2.** Representative FACS analyses of yeast strains to verify G1 arrest.

## Data Availability

The data that support the findings of this study are available in figures and the supplementary material of this article. Raw data are available from the corresponding author at aha@mbg.au.dk upon reasonable request.
