# Supplementary figures and images for: A cellular system to study responses to a collision between the transcription complex and a protein‐bound nick in the DNA template
Source: FEBS Lett. 2025 May 1;599(12):1749–59. doi: 10.1002/1873-3468.70053 (PMC12183625; doi:10.1002/1873-3468.70053)

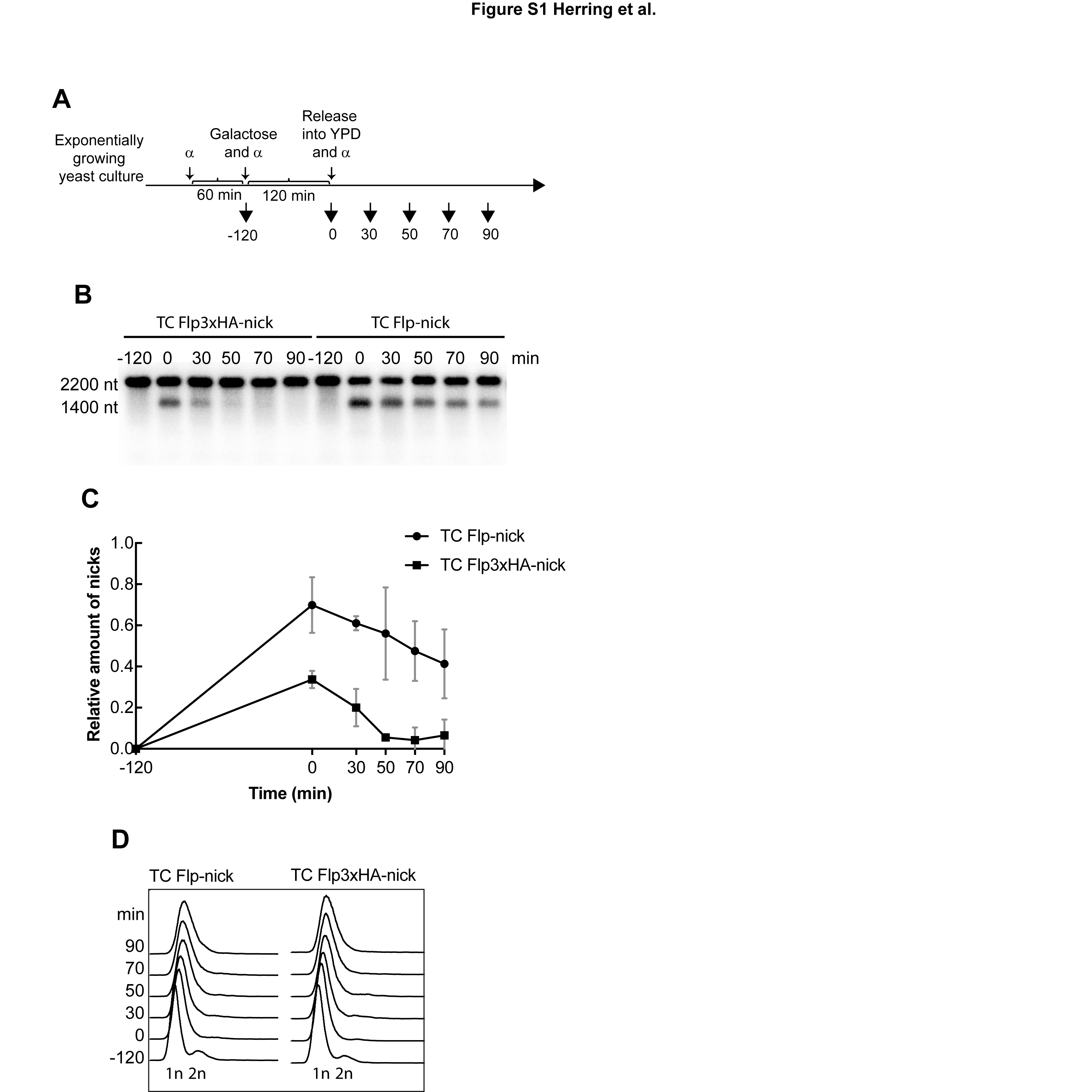

Supplement: Supplementary file 1 — Fig. S1. The 3xHA tag reduces the cleavage activity/stability of FlpH305L. [file FEB2-599-1749-s002.tif]

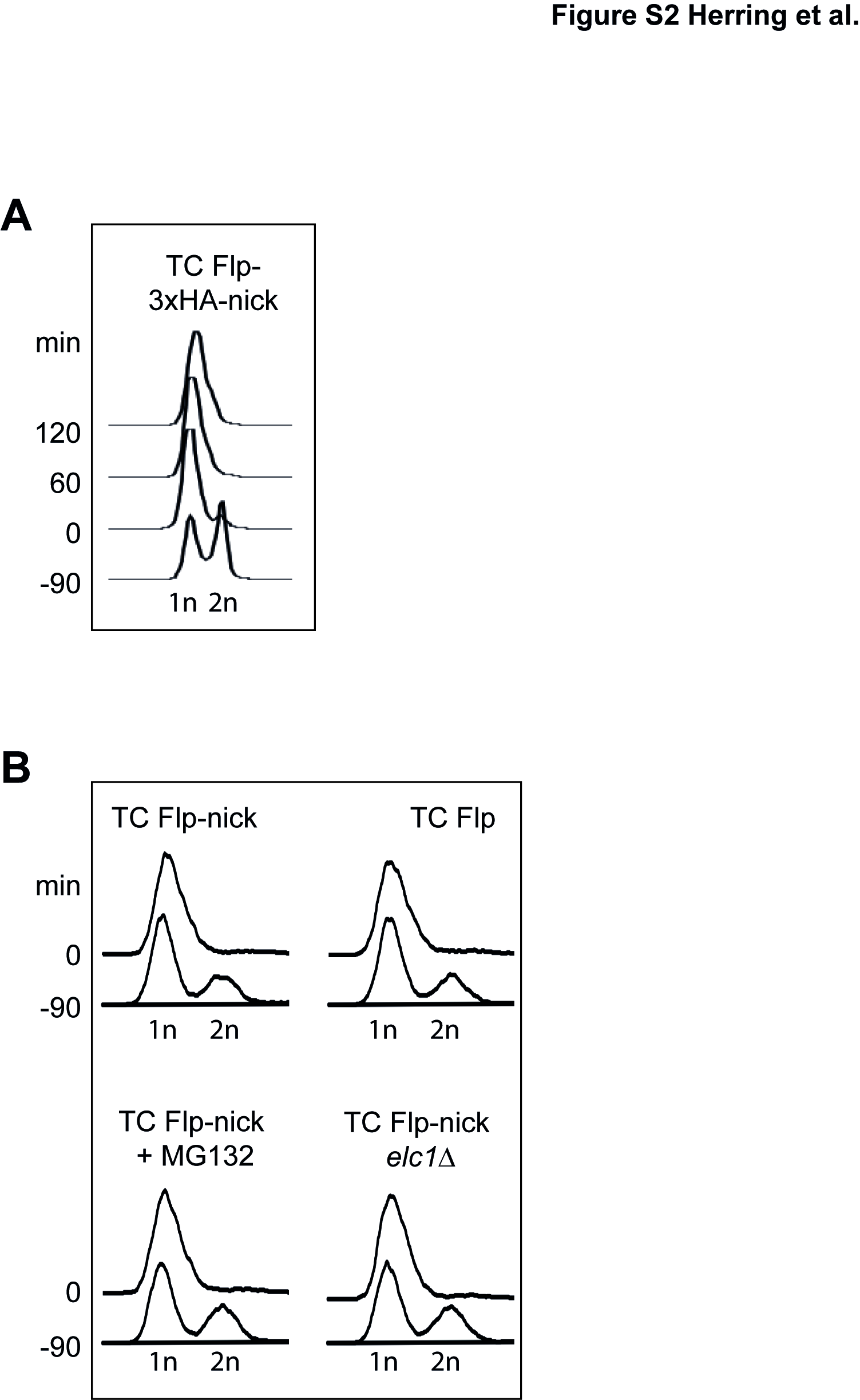

Supplement: Supplementary file 2 — Fig. S2. Representative FACS analyses of yeast strains to verify G1 arrest. [file FEB2-599-1749-s001.tif]
